# Depression and its correlations with health-risk behaviors and social capital among female migrants working in entertainment venues in China

**DOI:** 10.1371/journal.pone.0191632

**Published:** 2018-02-28

**Authors:** Qiaohong Yang, Don Operario, Nickolas Zaller, Wen Huang, Yanyan Dong, Hongbo Zhang

**Affiliations:** 1 Zhejiang Provincial Center for Disease Control and Prevention, Hangzhou, Zhejiang Province, China; 2 School of Public Health, Brown University, Providence, United States of America; 3 Fay W. Boozman College of Public Health, University of Arkansas, Fayetteville, United States of America; 4 Shaoxing City Center for Disease Control and Prevention, Shaoxing, Zhejiang Province, China; 5 Nantong City Maternal and Child Health Care Hospital, Nantong, Jiangsu Province, China; 6 School of Public Health, Anhui Medical University, Hefei, Anhui Province, China; UNITED STATES

## Abstract

**Objectives:**

Among the dramatic increased internal migration in China in past three decades, a considerable proportion of young females migrated to urban areas and found employment in “entertainment venues”, who may be vulnerable to psychological distress. This study examines the prevalence of depression and explores its associations with health-risk behaviors and social capital among this subgroup.

**Methods:**

358 female migrants were recruited from entertainment venues in a rapidly growing urban city in China. A survey which included measures of depressive symptoms, health-risk behaviors, social capital, and socio-demographic characteristics was administered. Multivariable logistic regression was conducted to identify the independent correlates of depression.

**Results:**

Of participants, 31.0% had clinically significant depressive symptoms (CES-D score ≥ 16). In multivariable models, greater likelihood of depressive symptoms was associated with working in massage centers/hotels (OR = 3.20, 95% CI: 1.80–5.70), having probable alcohol dependence (OR = 2.25, 95% CI: 1.22–4.16), self-reported lifetime use of illicit drugs (OR = 2.98, 95% CI: 1.26–7.06), growing up in a non-nuclear family (OR = 2.46, 95% CI: 1.18–5.16), and poor social capital (OR = 6.01, 95% CI = 2.02–17.87).

**Conclusion:**

Intervention strategies to address the high prevalence of depression among female migrants are needed, and should also aim to reduce problematic alcohol and drug use, improve social capital, and target women working in massage centers or hotels.

## Introduction

Stimulated by rapid modernization and industrialization, China has witnessed a dramatic increase in internal migration in the past three decades. The estimated number of internal migrants in China increased from 6.75 million in 1982 to 253 million in 2014 [1, 2]. Many rural-to-urban migrants in China seek economic opportunities for themselves and their families, however they often lead socially and residentially isolated lives in the city [3, 4]. due to the distance away from their family and the “Hukou” system (i.e., the Chinese system of household registration) which limits access to employment, education, housing, health care, and social services for those who are not permanent city residents [5, 6]. Mental health problems among rural-to-urban migrants in China who are often young working-age adults, due to geographic dislocation form family and communities of support are also an emerging concern. A nascent body of literature has reported more severe depressive symptoms among Chineseinternal migrants compared with the general population [7, 8]. Indeed, several highly publicized suicides and attempted suicides of young migrant workers in southern China brought widespread public attention to this population [9].

A study of internal migrants in five major cities in China revealed that females accounted for 56% of among young migrants between the ages of 15–29 years old [10]. Previous studies have found that female migrants in China are more vulnerable to psychological distress and poor mental health symptoms than their male counterparts [11, 12]. Because of low education levels and limited formal skills training, a substantial number of young female migrants undertake employment in entertainment venues [13, 14]. Entertainment venues are a heterogeneous set of commercial venues including large nightclubs and karaoke establishments where alcohol is serviced, as well as bath and body/foot massage establishments which are often located in or adjacent to hotels. Patrons (predominantly male) of these venues pay to engage in social and relaxation activities with employees (predominantly female) such as dancing, karaoke, gambling, and massage. Most services provided by women working in these venues are non-sexual in nature; however, some venues/employees also offer additional sexual services to clients for additional fees. Employees of these entertainment venues have significant exposure to alcohol and substance use due to the types of interactions with male patrons; these employees also may have elevated vulnerability for sexual risk such us commercial sex, multiple sexual partners, and unprotected sexual behaviors that might contribute to HIV and other sexually transmitted infections [15–17]. However, little is known about the prevalence and correlates of depression among employees of entertainment venues.

With the growing recognition of the social determinants of health, the concept of social capital has become an increasingly important concept to explain relationships between the social environment, social relationships, and mental health [18–21]. Although definitions of social capital vary across studies, most conceptualizations can be divided into two components: structural and cognitive. The structural component of social capital includes the extent and intensity of interpersonal links or associational activities that provide support to a person and potentially reduce the negative impacts of adverse life events. The cognitive component refers to subjective perceptions of support, reciprocity, sharing, and trust that may increase feelings of security and self-esteem and consequently improve psychological well-being [22, 23]. Social capital among migrant populations is frequently “ruptured” due to displacement from support networks, rendering migrant individuals vulnerable to social, psychological, and health risks [24]. Associations between low social capital and depression have been reported in previous studies [25–28]. In China, associations between social capital and depression have been documented in studies involving urban Chinese adolescents, the elderly, and rural children who have experienced parental absence [29–31]. A few studies of Chinese internal migrants have documented low levels of social capital [32–34].

Previous studies of Chinese female migrants have examined risk outcomes such as abortion, sexually transmitted infections, and HIV in China [14, 35], no known studies have reported on depression and its correlates with these risk outcomes or with social capital. The purposes of this study were to (a) describe the prevalence of depressive symptoms among female migrants working in entertainment venues in a city of China, and (b) explore the associations of depressive symptoms with health-risk behaviors and social capital. Findings from the present study will contribute a step towards characterizing mental health problems in this group, and can facilitate the design of intervention strategies to promote the psychological well-being among these female migrants.

## Methods

### Participants and recruitment

From March to July 2012 we conducted a cross-sectional study among young migrant women employed in entertainment venues in Hefei, the capital city of Anhui Province, China. The study site represents a rapidly growing urban setting in central China (estimated population of 7.8 million) and is developing into a national hub for science and technology industries, and is a site for migration for many people in rural central China who cannot or choose not to migrate to the larger mega-cities on China’s eastern coast. In general, migrants to Hefei are young (53% younger than 29 years old), seeking job opportunities (62%), and have low levels of education (78% have not completed high school) [36]. Eligibility included being female, ages 18 to 29 years old, a rural migrant (defined as someone born and raised in a rural area who does not have a Hefei hokou), and employed in an entertainment venue in Hefei. We recruited participants from entertainment venues in two districts in Hefei—Luyang district (a higher socioeconomic area) and Baohe district (a lower socioeconomic area). Prior to recruitment, we obtained a list of all 157 registered entertainment venues from the local administrative authority in both districts, where entertainment venues must be legally registered. We randomly selected 56 entertainment venues from this list, and contacted the owners/managers of these venues their permission to recruit on site. We obtained permission from 48 entertainment venues. To avoid disrupting their business activities, we conducted participant recruitment prior to the beginning of the evening shifts by approaching female employees in the employee lounges and private dressing rooms and given them a study informational flier. The study was approved by Institutional Review Boards at Anhui Medical University and Brown University.

### Procedure

Individuals who expressed initial interest were scheduled to meet outside of work hours in a private location to conduct the survey. After confirming their eligibility and providing informed consent, participants completed a structured survey which was administered verbally by a trained staff member; research staff (comprising a team of young women) received training in non-coercing recruitment, ethics, consent procedures, and on strategies to minimize social desirability and maximize fidelity to study procedures. During screening, we enquired whether any women felt forced or coerced to participate, and we planned to exclude these women from participation; no women reported force or coercion. Of 374 women who responded to outreach efforts and expressed an interest in the study, 16 did not meet eligibility criteria. A total of 358 women were enrolled and completed a structured questionnaire interview. The questionnaire took about 30–45 minutes to complete. Each participant was compensated with a small gift worth 50 Chinese Yuan (approximately 7.30 U.S. dollars) after completion of the survey, and all participants were informed about health services provided by the local CDC including sexual and reproductive health and mental health services.

### Measures

We assessed socio-demographic characteristics, including: age; types of workplace (categorized as karaoke parlors/nightclubs, massage centers/hotel); education level (junior high school or less, high school or more); marital status, family status (i.e., whether the participant was raised in a nuclear family, single-parent family, or step-parent family); monthly income (less or greater than 4000 RMB), and length of time working in Hefei (in years). We assessed sexual health and substance use behaviors using measured validated or administered previously in studies of migrants and vulnerable populations in China. Sexual behaviors assessed in this study were limited to vaginal sex; anal and oral sex behaviors were not assessed. We assessed whether participants engaged during the past 6 months in commercial sexual behavior, casual sexual behavior (i.e., outside of a committed relationship), sex with multiple partners. Condom use with each sexual partner type (husband, male clients, boyfriend, and casual partners) in the previous 6 months was assessed. Alcohol use was measured using the Alcohol Use Disorder Identification Test (AUDIT) [37]; a score ≥ 16 was defined as probable alcohol dependence. In the absence of a unified measure of social capital [38], we assessed social capital using an exploratory 4-item measure that inquired whether participants had satisfying levels of social participation, fair access to health services, trust in the social environment, and social support in their current lives in Hefei; response options to each item were “Yes” or “No” and items were summed to achieve a 0–4 scale. Depressive symptoms were assessed using the Center for Epidemiologic Studies Depression Scale (CES-D) [39]. A cutoff score of 16 was used to define a clinically significant level of depressive symptoms [40]. Use of this measure and the cutoff score have been validated elsewhere in China [41, 42].

### Analysis

We conducted descriptive analyses for socio-demographic characteristics and levels of depressive symptoms, health-risks, and social capital. Pearson Chi-square tests were employed to explore the associations of depressive symptoms (above or below the 16-score cutoff) with socio-demographic characteristics, health risks, and social capital. We conducted multivariable logistic regression to identify the independent correlates of depressive symptoms. We entered variables into the model if they had a moderate bivariate association with depressive symptoms (*p* < 0.10) based on procedures described in Hosmer and Lemeshow [43]. Data were entered using EpiData 3.0 and all statistical analyses were performed using SPSS 10.01.

## Results

A total of 358 participants met inclusion criteria and completed the survey. Socio-demographic data are shown in Table 1. Approximately two-thirds (65.1%) of the participants were recruited from karaoke parlors and nightclubs and about one-third (34.9%) were recruited from massage centers. The mean age was 23.5 years (SD = 2.9). Over half (55.6%) had attained a junior high school education or less, and nearly 45.0% were married or living with a boyfriend. Most participants (89.7%) had grown up in an intact family of both parents married and living together. About half (51.7%) had earned more than 4000 Yuan (roughly 640 USD) monthly. Seventy percent had been working as a migrant for more than 2 years (SD = 2.9).

**Table 1:** Demographic characteristics and depressive symptoms among participants.

Variables	N(%)	CES-D≥16	CES-D <16	*χ*^*2*^	*P/t*-value
		n(%)	n(%)		
Types of venues					
Karaoke parlors/nightclubs	233(65.1)	62 (26.6)	171(73.4)	6.03	0.014
Massage centers/hotels	125(34.9)	49 (39.2)	76(60.8)		
Age (years)					
18–20	66 (18.4)	27 (40.9)	39(59.1)	3.73^a^	0.053
21–25	196(54.7)	59 (30.1)	137(69.9)		
26–29	96 (26.8)	25 (26.0)	71(74.0)		
Education Level					
Junior high school or less	199(55.6)	71(35.7)	128(64.3)	4.57	0.032
High school or higher	159(44.4)	40 (25.2)	119(74.8)		
Marital status					
Single/divorced or widowed	198(55.3)	57 (28.8)	141(71.2)	1.02	0.313
Married/living with boyfriend	160(44.7)	54 (33.8)	106(66.2)		
Grown up in a nuclear family					
Yes	321(89.7)	93 (29.0)	228(71.0)	6.00	0.014
No	37 (10.3)	18 (48.6)	19(51.4)		
Monthly income					
≤ 4000	173(48.3)	52 (30.1)	121(69.9)	0.14	0.708
> 4000	185(51.7)	59 (31.9)	126(68.1)		
Migrant work time (years)					
≤ 2	108(30.2)	31 (28.7)	77(71.3)	0.38	0.536
> 2	250(69.8)	80 (32.0)	170(68.0)		

^a^ Tested using Linear-by-Linear Association Chi-Square Tests

Overall, 31.0% (111/358) of participants had clinically significant depressive symptoms (CES-D score ≥ 16; range = 0–46, M = 12.9, SD = 9.0). Correlations between participant characteristics and depressive symptoms are reported in Table 1. Compared with non-depressed participants, those with clinically significant depressive symptoms were more likely to have worked in massage centers, to have received a junior high school or lower level of education level, and to have grown up in a non-nuclear family (i.e., single-parent or step-parent family).

As shown in Table 2, Participants who had clinically significant depressive symptoms were more likely to have had unprotected sex (defined as ever having vaginal sex without a condom) with clients or casual sexual partners in previous 6 months, to have probable alcohol dependence (AUDIT ≥ 16), and to have ever used illicit drugs (Table 2).

**Table 2:** Health-risk behaviors and associations with depressive symptoms.

Variables	N(%)	CES-D≥16	CES-D <16	*χ*^*2*^	*P/t*-value
		n(%)	n(%)		
Had commercial sexual behaviors in the past 6 months					
Yes	154 (43.0)	48 (31.2)	106 (68.8)	0.00	0.95
No	204 (57.0)	63 (30.9)	141 (69.1)		
Multiple sexual partners in past 6 months					
Yes	149 (41.6)	51 (34.2)	98 (65.8)	1.24	0.27
No	209 (58.4)	60 (28.7)	149 (71.3)		
Had any unprotected sexual behaviors in past 6 months					
Yes	219 (61.2)	76 (34.7)	143 (65.3)	3.61	0.06
No	139 (38.8)	35 (25.2)	104 (74.8)		
Had unprotected sexual behaviors with husband/boyfriend in past 6 months					
Yes	205 (57.3)	67 (32.7)	138 (67.3)	0.63	0.43
No	153 (42.7)	44 (28.8)	109 (71.2)		
Had unprotected sexual behaviors with clients/casual sexual partners in past 6 months					
Yes	32 (8.9)	16 (50.0)	16 (50.0)	5.93	0.02*
No	326 (91.1)	95 (29.1)	231 (70.9)		
Probable alcohol dependence (AUDIT ≥ 16)					
Yes	95 (26.5)	37 (38.9)	58 (61.1)	3.82	0.05*
No	263 (73.5)	74 (28.1)	189 (71.9)		
Ever used illicit drugs					
Yes	27 (7.5)	15 (55.6)	12 (44.4)	8.23	<0.01*
No	331 (92.5)	96 (29.0)	235 (71.0)		

* *p* < 0.05

Participants’ endorsements of the social capital statements are shown in Table 3. Overall, 58% reported having satisfying levels of social participation, 79.9% reported having fair access to social services in Hefei, 45.8% reported having good trust in the social environment, and 51.1% reported having good social support in Hefei. Participants with clinically significant depressive symptoms were more likely to have reported poor social participation and unfair public health services. Of note, less than one-fifths (19.3%) reported a high degree of social capital with all four items, and lower levels of social capital were associated with depressive symptoms (Table 3).

**Table 3:** Social capital and associations with depressive symptoms.

Variables	N(%)	CES-D≥16	CES-D <16	*χ*^*2*^	*P/t*-value
		n(%)	n(%)		
Had satisfying social participation in the city					
Yes	208 (58.1)	52 (25.0)	156 (75.0)	8.37	<0.01*
No	150 (41.9)	59 (39.3)	91 (60.7)		
Had fair access to health services in the city					
Yes	286 (79.9)	74 (25.9)	212 (74.1)	17.5	<0.01*
No	72 (20.1)	37 (51.4)	35 (48.6)		
Had trust in the social environment of the city					
Yes	164 (45.8)	47 (28.7)	117 (71.3)	0.78	0.38
No	194 (54.2)	64 (33.0)	130 (67.0)		
Had good social support in the city					
Yes	183 (51.1)	53 (29.0)	130 (71.0)	0.73	0.39
No	175 (48.9)	58 (33.1)	117 (66.9)		
Level of social capital					
4 of 4	69 (19.3)	15 (21.7)	54 (78.3)	11.60^a^	<0.01*
3 of 4	98 (27.4)	26 (26.5)	72 (73.5)		
2 of 4	102 (28.5)	32 (31.4)	70 (68.6)		
1 of 4	67 (18.7)	24 (35.8)	43 (64.2)		
0 of 4	22 (6.1)	14 (63.6)	8 (36.4)		

^a^ Tested using Linear-by-Linear Association Chi-Square Tests

* *p* < 0.05

Table 4 shows the variables that had significant associations with depressive symptoms in the multivariable logistic regression model. Greater odds of depressive symptoms were associated with working in a massage centers (OR = 3.20, 95% CI: 1.80–5.70), having grown up in a non-nuclear family (OR = 2.46, 95% CI: 1.18–5.16), having probable alcohol dependence (OR = 2.25, 95% CI: 1.22–4.16), self-reported lifetime use of illicit drugs (OR = 2.98, 95% CI: 1.26–7.06), and poor social capital (OR = 6.01, 95% CI: 2.02–17.87).

**Table 4:** Multivariable logistic regressions: Correlates of depressive symptoms.

Variables	Univariate regression		Multivariable regression	
	OR (95% CI)	*P*-value	AOR (95% CI)	*P*-value
Types of venues				
KTVs/nightclubs	1		1	
Massage centers	1.78 (1.12–2.82)	0.02	3.20 (1.80–5.70)	<0.01
Age (years)				
26–29	1			
21–25	1.22 (0.71–2.12)	0.47		
18–20	1.97 (1.01–3.84)	0.05		
Education Level				
High school or higher	1			
Junior high school or less	1.65 (1.04–2.62)	0.03		
Grown up in a nuclear family				
Yes	1		1	
No	2.32 (1.17–4.62)	0.02	2.46 (1.18–5.16)	0.02
Had any unprotected sexual behavior in past 6 months				
No	1			
Yes	1.68 (0.98–2.88)	0.06		
Had unprotected sexual behavior with clients/casual sexual partners in past 6 months				
No	1			
Yes	2.43 (1.17–5.06)	0.02		
Probable alcohol dependence				
No	1		1	
Yes	1.63 (1.00–2.67)	0.05	2.25 (1.22–4.16)	0.01
Ever used illicit drugs				
No	1		1	
Yes	3.06 (1.38–6.78)	0.01	2.98 (1.26–7.06)	0.01
Social capital level				
4 of 4	1		1	
3 of 4	1.30 (0.63–2.69)	0.48	1.27 (0.59–2.72)	0.54
2 of 4	1.65 (0.81–3.34)	0.17	1.65 (0.78–3.47)	0.19
1 of 4	2.01 (0.94–4.29)	0.07	2.04 (0.92–4.50)	0.08
0 of 4	6.30 (2.23–17.83)	0.01	6.01 (2.02–17.87)	<0.01

## Discussion

Given trends in rural-to-urban migration, modernization, and social-economic development in China, emerging health challenges related to the mobile status and living/working situations of migrant populations warrant examination. The current study adds to a growing body of research on rural-to-urban female migrants in China [7,8,10,12,13], by bringing attention to the prevalence of depressive symptoms and the correlation between depression, health-risk behaviors, and social capital among young female migrants working in entertainment venues. Our results indicated that 31% of women in our study had clinically significant depressive symptoms. Findings from this study show a need for more attention toward understanding and addressing depression and mental health needs in this marginal and vulnerable population.

Due to the social conditions and employment expectations of entertainment venues, female migrants working in these venues have high exposure to and risk for engagement in hazardous alcohol use, sexual risk behaviors, and illicit drug use [13–17]. The additional observation of depressive symptoms reported in this paper give evidence to a “syndemic” among this group—which has been defined previously as the co-occurrence of multiple health problems within a specific population [44]. According to syndemic theory, co-occurring health problems (e.g., depression, substance use, and HIV risk) interact with and reinforce one another, and may give rise to additional health problems within the population. Syndemics are often the downstream consequences of structural and systemic factors that produce a context for negative health within disadvantaged communities. Consequently, patterns of syndemic health problems must be examined within their social context. We found that female migrants working in entertainment venues who reported probable alcohol dependence and illicit drug use were also more likely to have clinically significant depressive symptoms, which suggests that further interventions aimed to reduce depressive symptoms should take into consideration the alcohol and illicit drug use among this population. Depression also was associated with condomless sex casual and commercial partners during the past 6 months. In accordance with syndemic theory, efforts to address any of these health conditions must also address the co-occurrence of other health conditions present among female migrants working in entertainment venues, as well as acknowledge the social conditions that uniquely render this population vulnerable to multiple health problems [45].

Although economic factors are widely acknowledged as primary drivers of rural-to-urban migration in China, there is a need for further research to explore more fully the range of social, familial, personal, as well as economic drivers of migration and potential determinants of depression for young women who work in entertainment venues. Two demographic characteristics were correlated with clinically significant depressive symptoms in this study. First, compared with women working in karaoke parlors or nightclubs, those working in massage centers or hotels had three-fold greater odds of having clinically significant depressive symptoms. Reasons for this association are unclear, but may be due in part to the lower status of these venues and higher vulnerability for engagement in commercial sex work compared with working in karaoke and nightclub settings, which may contribute to increased depressive symptoms. Female migrants who work in massage centers and hotels might also have different migration trajectories into these employment venues compared with those who work in karaoke bars or nightclubs, and the working conditions (e.g., regard/treatment by management, environmental settings, types of labor performed) between these employment venues might also differ in ways that contribute to employees’ depression. Although this finding demands further research, it suggests that massage centers and hotels warrant particular attention for venue-based interventions that aim to reduce depressive symptoms of female migrant workers. Second, female migrants who grew up in a non-nuclear family were more likely to have clinically significant depressive symptoms. This finding corresponds with other research showing that emotional distress or health-risk behaviors are more prevalent among children and adolescents from single-parent families or step-families compared with those growing up in nuclear families [46, 47]. This finding also suggests that non-economic factors might drive migration for some young women in China—e.g., due to the desire to separate from families of origin and establish personal independence—and contribute to overall mental health. More in-depth analysis (e.g., using qualitative methodologies) can provide more complex insight into the range of trajectories and motivations for migration and the concomitant health implications of different migratory motives.

Poor levels of social capital were revealed in this sample, with approximately half of the participants reporting having no satisfying social participation, social trust, or social support in the city, and less than 20% reporting all four social capital indicators. These findings might reflect the working conditions and social status of these women. Due to institutional barriers associated with the Hukou system, migrants have limited access to services and socioeconomic opportunities in their new city of residence. This issue highlights a structural tension and a potential health risk in the motive for migration—that despite the desire for new economic opportunities in larger urban cities, migrants face exclusion from services and legitimate opportunities for advancement due to their non-permanent Hukou status. Employment in entertainment venues can bring further social problems due to the perception that these work venues are sites for commercial sex work [13]. Consequently, many female migrants working in entertainment venues do not report their occupation to family members and friends [48], which may exacerbate weakened family support during the migration process [4, 49]. Over time, these factors may contribute to depressive symptoms among migrant women working in entertainment venues. Indeed, in the multivariable models, women with the lowest levels of social capital had significantly higher odds of depressive symptoms. However, as stated earlier, this cross-sectional statistical association might reveal deeper patterns and more complex trajectories of family, personal, and economic background characteristics that drive migration, and their concomitant implications for social capital and mental health.

Several limitations to this research should be considered. First, due to the stigmatized and marginalized status of female migrants working in entertainment venues in China, our data were subject to volunteer bias and socially desirable reporting bias. Second, our data were also subject to recall bias, as participants were asked to recall their previous sexual behaviors during the past six months. Third, due to the non-representative sampling methods and geographic recruitment settings, findings from this study might not be generalizable to female migrants elsewhere in China. Fourth, information on additional factors that can contribute to depression among female migrants, such as discrimination and housing conditions, were not assessed. Due to sample size restrictions and restricted range, we were unable to test moderators of the association of social capital on depression (e.g., age, socioeconomic background) which might reveal specific population subtypes with greater risk. Fifth, the measure of social capital was exploratory and findings from this measure must be interpreted with caution. Additional measurement development is needed to refine strategies for assessing social capital in China. Finally, we cannot make casual or temporal interpretations due to the nature of cross-sectional data. Other research designs, such as longitudinal cohort studies, and more complex measurement and analytic strategies are needed to understand syndemic processes by which depressive symptoms are associated and determined by co-occurring health risks behaviors, social capital, and “upstream” social factors that contribute to health vulnerability in these women.

In conclusion, the current study contributes additional knowledge toward understanding the prevalence of depression and its co-occurrence with health-risk factors and social capital among female migrants working in entertainment venues in China. Our study underscores the high prevalence of depressive symptoms and call for more attentions on depressive symptoms and mental health needs of this marginal and vulnerable population. Female migrants working in massage centers or hotels have higher prevalence of depressive symptoms, which implies that mental health interventions targeting these workplaces are needed. Potential intervention strategies may also focus on addressing alcohol and illicit drug use and increasing levels of social capital among female migrants working in entertainment venues. On a structural level, efforts to reform the Hukou system, in order to reduce inequalities between urban and rural populations, and to encourage entertainment venue owners/managers to support the social integration and well-being of female migrant workers may benefit this population. Future research and mobilization efforts are needed to understand factors that can motivate entertainment venue owners/managers to participate in public health efforts for supporting the well-being of their employees.

## Supporting information

S1 Dataset Relevant data underlying the findings described in manuscript. (SAV)
